# Microbial Organic Fertilizer Combined with Magnetically Treated Water Drip Irrigation Promoted the Stability of Desert Soil Aggregates and Improved the Yield and Quality of Jujubes

**DOI:** 10.3390/plants13141930

**Published:** 2024-07-12

**Authors:** Wanghai Tao, Fanfan Shao, Haokui Yan, Quanjiu Wang

**Affiliations:** State Key Laboratory of Eco-Hydraulics in Northwest Arid Region, Xi’an University of Technology, Xi’an 710048, China; xautsoilwater@163.com (W.T.); shaoffan@126.com (F.S.); hky01222@163.com (H.Y.)

**Keywords:** jujube, microbial organic fertilizer, enzyme activities, soil aggregate stability, growth, yield

## Abstract

In the southern Xinjiang region of China, developing efficient irrigation and fertilization strategies to enhance resource utilization and prevent desertification is of critical importance. This study focuses on jujubes in Xinjiang, China, and involves a three-year field experiment aimed at exploring the optimal application strategy of magnetically treated water combined with microbial organic fertilizer to provide scientific support for high-quality jujube production. The experiment included a control group (using only fresh water, denoted as CK) and combinations of magnetically treated water drip irrigation with varying amounts of microbial organic fertilizer: in 2021, treatments included M0 (only irrigating with magnetically treated water), M6 (0.6 t/ha), M12 (1.2 t/ha), M18 (1.8 t/ha), and M24 (2.4 t/ha); in 2022 and 2023, treatments included M0, M6 (0.6 t/ha), M12 (1.2 t/ha), M24 (2.4 t/ha), and M48 (4.8 t/ha). This study investigated the effects of magnetically treated water drip irrigation combined with microbial organic fertilizer on soil physical properties, hydraulic parameters, enzyme activity, aggregate stability, and jujube yield and quality. The application of microbial organic fertilizer significantly reduced the soil bulk density by 3.07% to 11.04% and increased soil porosity by 1.97% to 14.75%. Soil saturated hydraulic conductivity gradually decreased with the increasing amount of microbial organic fertilizer, with a reduction range of 5.95% to 13.69%, while the water-holding capacity significantly improved (from 0.217 cm^3^/cm^3^ to 0.264 cm^3^/cm^3^). Additionally, microbial organic fertilizer significantly enhanced the activities of urease, catalase, and sucrase in the soil and significantly increased the proportion of large soil aggregates. Jujube yield increased by 3.66% to 21.38%, and the quality significantly improved, as evidenced by the increase in soluble sugar and flavonoid content. The Gauss model calculation results recommended 3.09 t·hm^2^ as the optimal amount of microbial organic fertilizer for comprehensively improving jujube yield and quality. These findings indicate that magnetically treated water drip irrigation combined with high amounts of microbial organic fertilizer significantly improved soil physical properties, hydraulic parameters, enzyme activity, aggregate stability, and jujube yield and quality, providing scientific evidence for desert soil improvement and agricultural production.

## 1. Introduction

The impacts of global warming and the expansion of human activities have intensified climate and environmental challenges, such as extreme droughts, heavy rainfall, urban waterlogging, and water pollution, significantly constraining the sustainable use of regional water and soil resources [1,2]. The efficient use of agricultural water resources is especially critical in extremely arid regions [3]. Simultaneously, soil desertification, which reduces soil productivity and cultivable land area, poses a major challenge to global agriculture [2,4]. Xinjiang, the largest province in China in terms of desertified and sandy land areas, is a crucial agricultural region in the arid northwest. The province has 1.0686 million square kilometers of desertified land and 0.7468 million square kilometers of sandy land [5]. More than half of the land in southern Xinjiang is desertified [6]. In particular, the desertification of sandy soils along the southern edge of the Taklamakan Desert severely degrades soil quality, directly affecting agricultural sustainability in the region [7].

Jujubes (Zizyphus jujuba Mill.), one of China’s traditional fruits, hold significant nutritional and medicinal value [8]. China is the world’s largest producer of jujubes, with a production of 7.73 million tons in 2020, accounting for 76% of the global total [9]. Xinjiang is the largest high-quality jujube production base in China, contributing more than half of the national output [10]. The region’s ample sunshine and significant diurnal temperature variation are conducive to the growth of jujube trees [11]. Xinjiang has a jujube planting area of 320,000 hectares, with 80% of it located around the Taklamakan Desert in southern Xinjiang [12]. However, the cultivated soil in southern Xinjiang is mostly desert soil, characterized by extremely low organic matter content and poor water and nutrient retention. This results in low irrigation water and fertilizer efficiency and subsequently low agricultural productivity. Therefore, implementing effective measures to improve water and fertilizer use efficiency and enhance the water and nutrient retention capacity of desert soils is crucial for the rational utilization of the limited water and soil resources in this region.

Magnetically treated water is a potentially advantageous technique for enhancing plants’ utilization of irrigation water. This type of water undergoes structural and property changes when exposed to a magnetic field, which increases its activity and offers benefits in ameliorating saline-alkali soils and promoting plant growth and development [13]. Studies have shown that magnetic and electric activation techniques reduce the contact angle of water on platinum surfaces, thereby increasing the ability of water to penetrate micropores. When water passes through a magnetic field, the average distance between water molecules increases, weakening or even breaking some hydrogen bonds within large molecular clusters, forming smaller clusters [14,15,16]. The number of free monomer water molecules and dimers increases [14], osmotic pressure and solubility improve, and surface tension decreases [15]. Magnetically treated water significantly improves the distribution characteristics of soil pores, primarily by increasing the number of effective pores. The proportion of large pores increases from 8.8% to 14.8%, and the proportion of micro-pores increases from 13.1% to over 22.7% [15]. The effectiveness of magnetically treated water can last for about 14 days. Compared to conventional water irrigation, magnetically treated water irrigation reduces soil pH by 2.23%, decreases the soil bulk density by 1.71%, and increases soil porosity by 1.34% [16]. Additionally, it enhances the permeability of loess soil and accelerates the release of available nitrogen [17]. Besides its significant impact on improving the soil characteristics in the crop root zone, magnetically treated water also demonstrates excellent performance in promoting crop growth. Experimental results from the Xinjiang region indicate that under magnetically treated water drip irrigation, cotton yield and water productivity increased by 28.8% and 27.4%, respectively [18]. Furthermore, magnetically treated water increased the chlorophyll, protein, and soluble sugar content in tomato plants [19]. It also enhanced photosynthesis, raising the net photosynthetic rate of grape leaves and lettuce by 6.06% and 14.73%, respectively [20,21]. Magnetically treated water significantly increased the chlorophyll a, chlorophyll b, and carotenoid contents in poplar seedlings by 16.99%, 8.67%, and 17.32%, respectively, compared to conventional water [22].

Numerous studies have demonstrated that the application of microbial organic fertilizers can significantly improve the growth and development of field crops by optimizing nutrient absorption and utilization, thereby reducing dependence on chemical fertilizers [23,24,25,26]. Specifically, microbial organic fertilizers not only enhance the growth and capacity of crop root systems but also bring significant agricultural production benefits. For instance, the yield and growth of cotton crops have increased by up to 30% [23]. The application of microbial organic fertilizers effectively delays the coloration of the peel and flesh of blood oranges, increases the concentrations of nitrogen and potassium in leaves, and reduces titratable acidity (TA) [24]. When used as a base fertilizer, microbial organic fertilizers can increase the available phosphorus and potassium content in the soil, significantly enhancing wheat plant height, the number of grains per spike, and yield [25]. Notably, microbial organic fertilizers have a significant impact on soil microbial communities. During the wheat growth period, the relative abundance of Actinobacteria and Chloroflexi increases, while the relative abundance of Acidobacteria and Bacteroidetes decreases. The relative abundance of Proteobacteria first increases and then decreases, showing a closer relationship with the wheat growth stages [26].

Although magnetically treated water and microbial organic fertilizers have shown significant effects in improving soil environment, promoting crop growth, and enhancing crop quality, studies on the combined use of these treatments for soil improvement and growth promotion are still relatively rare [27]. Additionally, the mechanisms and long-term effects of magnetically treated water combined with microbial organic fertilizers under different soil types and climatic conditions remain unclear. Therefore, there is a need for in-depth research on more crop types, particularly high-value crops, such as jujubes, to explore the effects of microbial organic fertilizers on their growth, yield, and quality.

Based on these considerations, this study aims to achieve three primary objectives: (1) evaluate the impact of magnetically treated water combined with microbial organic fertilizers on soil water retention capacity, soil enzyme activity, and soil aggregate; (2) assess the contribution of magnetically treated water combined with microbial organic fertilizers to jujube yield and quality; and (3) comprehensively evaluate the efficacy of magnetically treated water combined with microbial organic fertilizer application in soil enhancement and jujube tree growth. By accomplishing these research objectives, we aim to obtain a comprehensive understanding of the influence of magnetically treated water combined with microbial organic fertilizers on both soil and crops, thereby establishing a scientific foundation for the sustainable development of jujube cultivation in southern Xinjiang.

## 2. Materials and Methods

### 2.1. Experimental Area

This study was conducted from 2021 to 2023 in a jujube cultivation area at the 8th Company of the 224th Regiment, 14th Division of the Xinjiang Production and Construction Corps, located in Kunyu City (37°21′45″ N, 79°19′60″ E). This area is characterized by a typical continental desert climate with the following climatic features: an annual average temperature of 12.2 °C, a total annual sunshine duration of 2705.6 h, annual precipitation of only 35 mm, a frost-free period of 225 days, and an evaporation rate of 3008.9 mm.

Soil samples were collected from the root growth layer (0–80 cm) of jujube trees within the experimental plots. Soil bulk density at different depths was measured every 20 cm using the ring knife method. The collected soil samples from different layers were air-dried and sieved through a 2 mm mesh. Soil particle size distribution, including sand, silt, and clay content, was determined using a Malvern laser particle size analyzer (MS 2000). Soil texture was classified according to the USDA soil texture classification standards. Additionally, soil pH, available potassium, and available phosphorus contents were measured. Detailed results are presented in Table 1.

**Figure 1:**
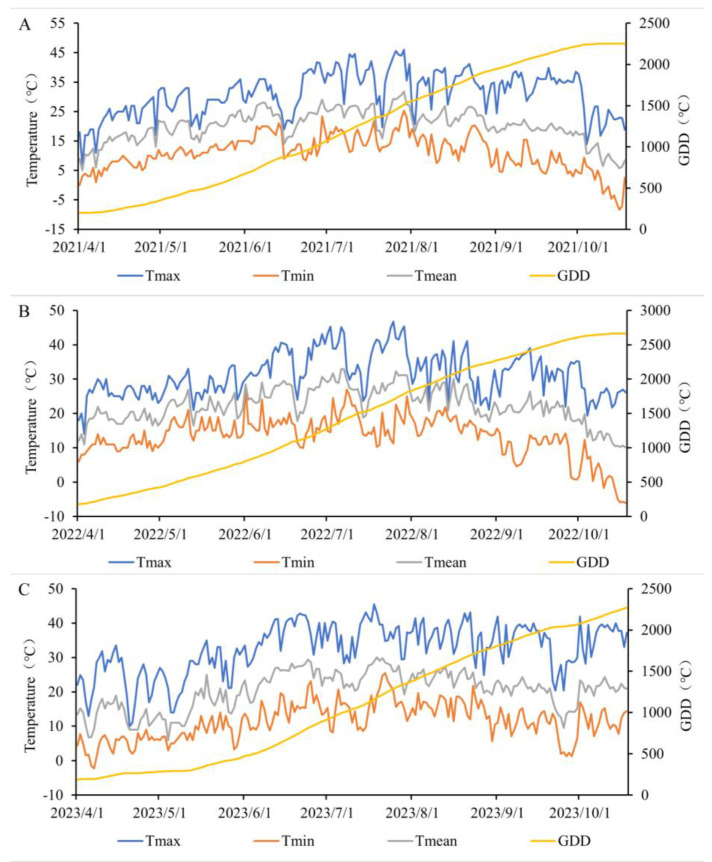
Temperature changes during the growing season of jujube tree in 2021 (**A**), 2022 (**B**), and 2023 (**C**).

A portable weather station recorded meteorological data every 30 min, including light intensity, photosynthetically active radiation, relative humidity, temperature, CO_2_ concentration, and rainfall. The average temperatures during the jujube growing seasons in 2021, 2022, and 2023 were 20.1 °C, 22.4 °C, and 20.3 °C, respectively (Figure 1). Rainfall amounts were 24.5 mm, 28.9 mm, and 33 mm, respectively. During the jujube growth periods, the average temperatures showed an initial increase followed by a decrease. The average temperature in April 2021 and April 2022 was around 15 °C, while in April 2023, it was around 10 °C. The average temperatures in April 2023 were lower than those in 2021 and 2022.

### 2.2. Experimental Design

#### 2.2.1. Irrigation and Fertilization Modes

**Figure 2:**
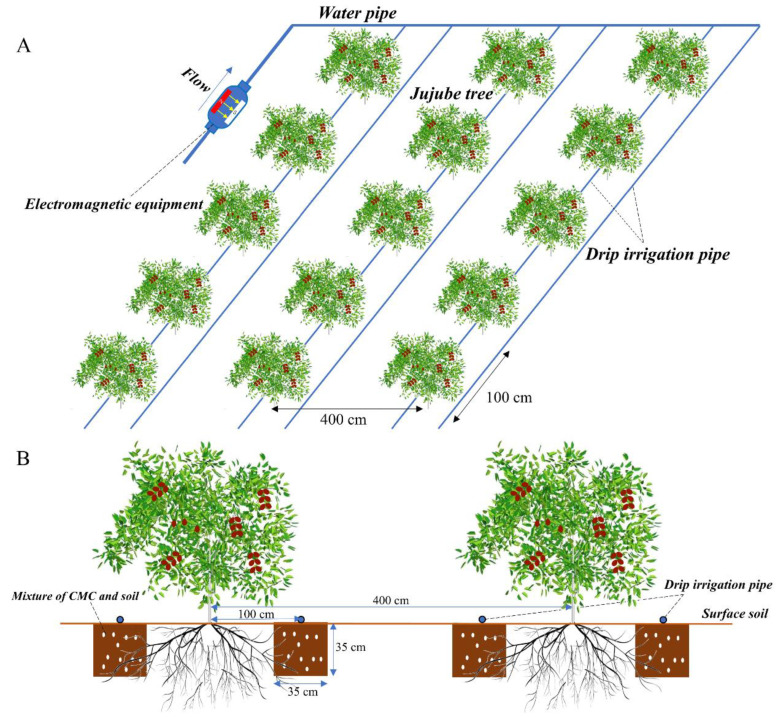
Jujube Planting Pattern (**A**) and Application Method of Microbial Organic Fertilizer (**B**).

The tested jujube trees were 12 years old with an open-center shape, planted using a dwarfing and high-density method. The average tree height was 2.5 m, with a spacing of 1.0 m between trees and 4.0 m between rows. Irrigation was conducted using a one-row two-pipe drip irrigation system (Figure 2). Each experimental plot was 4.0 m wide and 10.0 m long, containing a total of 10 jujube trees. To reduce water consumption and improve the quality of the jujubes, pinching and bud removal were carried out from May to July each year, along with pruning of new branches. Only one new tertiary branch was retained at the tip of each secondary branch, and all branches and fruit stalks on the primary branches were removed. Significant pruning was performed on the jujube trees from December to January each year to control tree growth, maintaining a tree height of 1.7 m and a canopy radius of 0.6 m.

The irrigation and fertilization regimes for 2021, 2022, and 2023 were shown in Table 2. The irrigation water, sourced from the melting snow and ice of the Kunlun Mountains, had a conductivity of 30 μs/cm, classifying it as fresh water. The total irrigation amount for both 2021 and 2022 was 320 mm. Fertilization was performed through fertigation, applying nutrients directly into the soil with the irrigation water. The total amounts of N (Urea, N 46%), P (P_2_O_5_, P 12%), and K (K_2_O, K 50%) applied were 405.75 kg/hm^2^, 166.50 kg/hm^2^, and 224.40 kg/hm^2^, respectively.

The irrigation water was magnetically activated using a magnetization device developed by Xi’an University of Technology. The magnetizer is equipped with a permanent magnet with a magnetic field strength of 3000 Gs. The water flow rate was controlled at approximately 6.0 m^3^/h through the magnetizer before entering the field pipelines. The layout of the irrigation system is shown in Figure 2.

#### 2.2.2. Experimental Design of Applying Microbial Organic Fertilizer under Drip Irrigation with Magnetically Treated Water

The microbial organic fertilizer used in this study was provided by Hubei Yanfan Group. The beneficial microbial communities primarily include Bacillus subtilis, Bacillus licheniformis, Bacillus amyloliquefaciens, actinomycetes, and yeast, with a total effective viable count of approximately 5 × 10^8^ CFU/g. The performance of the microbial organic fertilizer was determined according to the standard methods (NY 884-2012, Chinese Agricultural Industry Standard) [28]. The MOF had an organic matter content of 530 g/kg, a pH of 8.5, and total nitrogen content of 18.31 g/kg. It also contained 7.12% calcium, 6.69% silicon, 6.27% potassium, 3.58% chlorine, 2.42% iron, 1.37% phosphorus, 1.31% sulfur, and 1.08% magnesium.

In 2021, the treatments included fresh water drip irrigation (CK), magnetically treated water drip irrigation without microbial organic fertilizer (M0), and magnetically treated water drip irrigation with microbial organic fertilizer at application rates of 0.6 (M6), 1.2 (M12), 1.8 (M18), and 2.4 t/hm^2^ (M24). In 2022 and 2023, to determine the optimal application rate of microbial organic fertilizer, the application rates were further increased to 4.8 t·hm^−2^. The treatments included fresh water drip irrigation (CK), magnetically treated water drip irrigation without microbial organic fertilizer (M0), and magnetically treated water drip irrigation with microbial organic fertilizer at application rates of 0.6 (M6), 1.2 (M12), 2.4 (M24), and 4.8 t/hm^2^ (M48).

### 2.3. Quantitative Analysis of Indicators

#### 2.3.1. Determination of Soil Moisture Constants and Hydraulic Parameters

In the jujube planting area, soil samples were collected at depths of 5, 15, 30, 50, and 80 cm from the jujube tree planting zone, drip irrigation zone, and inter-row zone. Soil sampling was conducted using a handheld soil auger. The collected soil samples were then placed in an oven preheated to 105 °C and dried for 8 h. The soil moisture content was determined using the gravimetric method [12].

During the jujube harvest periods in 2021, 2022, and 2023, the top 0–10 cm of soil directly beneath the drip irrigation line was removed using a shovel. Soil samples were then collected from the 10–30 cm depth range using a ring knife to measure soil bulk density (BD) [14]. The ring knife containing the saturated soil was covered with filter paper to prevent evaporation and left in a cool place for 24 h to drain gravitational water, allowing for the determination of soil water-holding capacity (WHC). To measure soil saturated hydraulic conductivity (Ks), a new ring knife was placed on top of the ring knife containing the intact soil sample, and the two ring knives were sealed together with waterproof tape. Deionized water was poured into the new ring knife, and a graduated cylinder was placed below the ring knife with the soil sample to collect the outflow. The volume of liquid collected over a fixed period was recorded to calculate Ks [14]. Each soil sample was measured three times, and the average value was taken.

During the jujube maturation periods in 2021 and 2022, soil samples were collected from the 10–30 cm depth in the microbial organic fertilizer application area using a ring knife to measure soil bulk density (*BD*). Soil porosity was calculated using the following formula:

(1) $$SPV=1-\frac{BD}{PD}$$

where *PD* represents the particle density of the soil (=2.70 g/cm^3^).

A 4.0 g soil sample was placed into sieves with pore sizes of 2.0, 0.5, 0.25, and 0.125 mm. The sieves were immersed in water for 10 min and then shaken uniformly up and down for 10 min. The soil remaining on each sieve was then dried and weighed.

#### 2.3.2. Determination of Soil Nutrient Content and Enzyme Activity

Soil enzyme activity was determined following the methods described by Guan et al. (1986). Catalase activity (CE) in the soil was measured using the KMnO_4_ titration method [24,25]. In a 100 mL conical flask, 5.0 g of soil was mixed with 40 mL of distilled water and 5 mL of 0.3% H_2_O_2_ solution. The mixture was shaken for 20 min. Then, 5 mL of 3 mol·L^−1^ H_2_SO_4_ was added to the mixture, which was subsequently filtered. Finally, 25 mL of the filtrate was titrated with 0.1 mol·L^−1^ KMnO_4_ solution.

Urease activity (UE) was determined using a colorimetric method based on ammonium ion detection [14,15]. First, 3.0 g of soil sample was treated with 1 mL of toluene for 15 min and then mixed with 5 mL of urea solution (10%) and 10 mL of citrate buffer solution (pH 6.7). The mixture was incubated at 37 °C for 24 h. After incubation, the mixture was immediately filtered. The absorbance of the filtrate was measured at 578 nm using a spectrophotometer. A control without substrate was included for each sample. Urease activity was quantified as the amount of NH_3_-N produced per 1.0 g of air-dried soil.

The steps for determining sucrase activity (SE) are as follows: Take 2.0 g of soil sample and treat it with 1 mL of toluene. Then, mix it with 15 mL of 8% sucrose solution and 5 mL of phosphate buffer (pH 5.5). Incubate the mixture at 37 °C for 24 h, then filter it. Transfer 1 mL of the filtrate into a 50 mL volumetric flask and mix it with 3 mL of 3,5-dinitrosalicylic acid solution. Heat the mixture in a water bath for 5 min, then cool it for 10 min. Measure the absorbance of the filtrate at 508 nm using a spectrophotometer. Include a control without substrate for each sample [21].

#### 2.3.3. Jujube Yield and Quality Determination

In 2021, 2022, and 2023, during the jujube harvest period, all jujubes from the 10 trees in each plot were collected and weighed to calculate the yield. Four jujube samples were taken from the east, south, west, and north positions of each tree. The flavonoid content was determined using the aluminum nitrate-sodium nitrite colorimetric method. Soluble acids were measured using the acid–base neutralization transfer method, and soluble sugars were measured using the phenol-sulfuric acid method.

### 2.4. Gauss Model

The optimal application rate of microbial organic fertilizer and the corresponding optimal values for various indicators were calculated [16]. A four-parameter Gaussian optimization model was used to simulate the relationship between the application rate of microbial organic fertilizer and the target indicators. The calculation formula is as follows:

(2) $$TV=TV_{0}+a\times \exp\left(-\frac{{\left(C-C_{0}\right)}^{2}}{2\beta^{2}}\right)$$

where *TV* represents the target value, *TV*_0_ is the target value when the application rate of microbial organic fertilizer is zero, *a* is the maximum increase in the target value, *C* is the application rate of microbial organic fertilizer, *C*_0_ is the optimal application rate of microbial organic fertilizer, and *β* is the standard deviation. When *C* = *C*_0_, the optimal target value is obtained as *TV*_0_ + *a*.

### 2.5. Statistical Methods

The variance analysis was performed using SPSS software, version 25.0 (SPSS Institute, Inc., Cary, NC, USA). Fisher’s LSD (least significant difference) was used to detect differences between treatments, and the significant differences were determined by LSD at *p* < 0.05. All data represent an average of three replicates. Data processing was performed using EXCEL 2019, and data visualization and model calculations were carried out using Python 3.8.

## 3. Results and Discussion

### 3.1. Effects of Microbial Organic Fertilizer Applied through Magnetically Treated Water Drip Irrigation on Soil Physical Properties

#### 3.1.1. Soil Bulk Density

**Figure 3:**
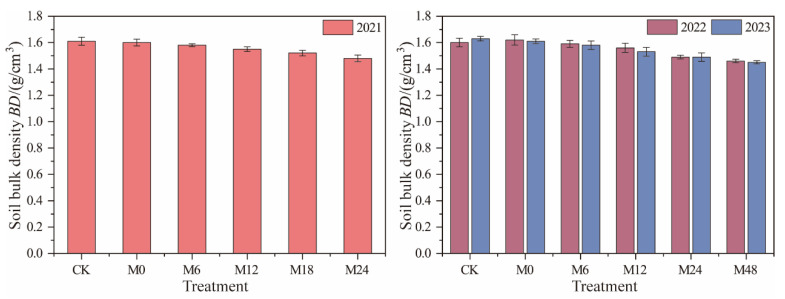
Changes in soil bulk density under magnetically treated water drip irrigation combined with microbial organic fertilizer.

Under magnetically treated water drip irrigation with microbial organic fertilizer, all treatments showed a reduction in the soil bulk density (BD) compared to the control (CK, BD = 1.61 g/cm^3^) in 2021 (Figure 3). The BD for treatments M6 to M24 decreased by 1.86% to 8.07%. This trend continued in 2022, with BD reductions ranging from 0.63% to 8.75% for treatments M6 to M48. In 2023, the BD of CK slightly increased to 1.63 g/cm^3^, while the BD of the microbial organic fertilizer treatments continued to decrease, with reductions ranging from 3.07% to 11.04% for treatments M6 to M48. Overall, the application of microbial organic fertilizer significantly reduced the soil bulk density, with the degree of reduction positively correlated with the amount of fertilizer applied. These results indicate that microbial organic fertilizer effectively reduces the soil bulk density by increasing the organic matter content and improving soil structure. As the amount of microbial organic fertilizer increased, the reduction in the soil bulk density became more pronounced. This could be because higher application rates of microbial organic fertilizer increase soil organic matter and microbial activity, which in turn improves the soil aggregate structure by enhancing the bonding between soil particles [15]. This aggregation forms larger soil clumps, reducing the soil bulk density and facilitating root expansion and gas exchange, thereby promoting plant growth [19].

#### 3.1.2. Soil Pore Volume

**Figure 4:**
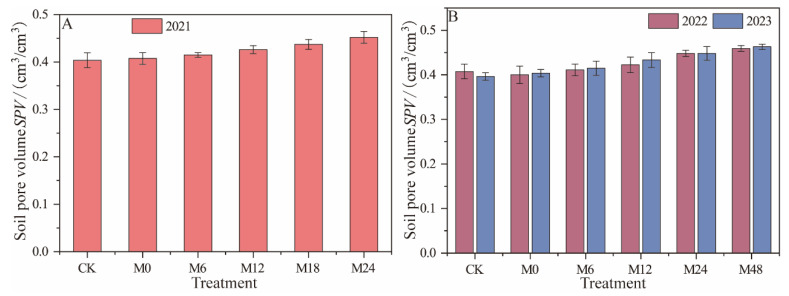
Changes in soil pore volume under magnetically treated water drip irrigation combined with microbial organic fertilizer (**A**,**B**).

Under magnetically treated water drip irrigation with microbial organic fertilizer (Figure 4), SPV (Soil Pore Volume) increased by 2.72% to 11.88% for treatments M6 to M24 compared to CK in 2021 and by 1.97% to 11.06% compared to M0, indicating a significant improvement in SPV due to microbial organic fertilizer. In 2022, the SPV of CK was 0.407 cm^3^/cm^3^. Among the microbial organic fertilizer treatments, M48 showed the highest increase in SPV, reaching 0.459 cm^3^/cm^3^, which was 12.78% and 14.75% higher than CK and M0, respectively, demonstrating the positive long-term effect of microbial organic fertilizer on soil porosity. In 2023, the treatments with microbial organic fertilizer continued to show an increase in SPV, especially at higher application rates. The SPV of the M48 treatment reached the highest value of 0.463 cm^3^/cm^3^, which was 16.92% and 14.60% higher than CK and M0, respectively, further confirming the long-term contribution of microbial organic fertilizer to enhancing soil porosity. Microbial organic fertilizer improves the physical structure of the soil by increasing organic matter content and microbial activity. These microbes not only decompose organic matter in the soil, producing colloidal substances that help soil aggregation, but also increase the proportion of curved macropores in desert soils through their biological activity [18]. These pores are crucial for the flow of water and air, improving the soil’s water retention capacity and the rhizosphere environment. As the application rate of microbial organic fertilizer increases, particularly at higher rates, this improvement becomes more pronounced, enhancing soil quality and promoting plant growth.

### 3.2. Effects of Microbial Organic Fertilizer Applied through Magnetically Treated Water Drip Irrigation on Soil Hydraulic Parameters

#### 3.2.1. Saturated Hydraulic Conductivity

**Figure 5:**
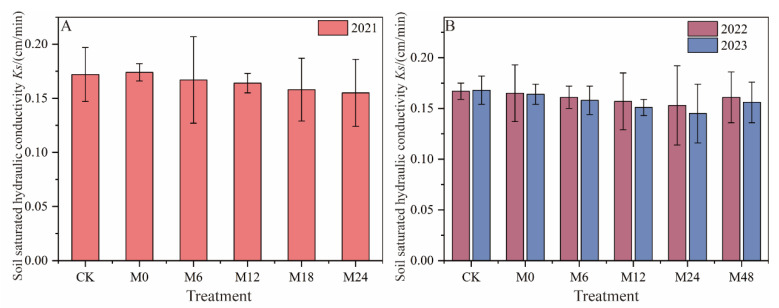
Changes in saturated hydraulic conductivity under magnetically treated water drip irrigation combined with microbial organic fertilizer (**A**,**B**).

Under magnetically treated water drip irrigation with microbial organic fertilizer, the saturated hydraulic conductivity (Ks) showed a decreasing trend with increasing application rates of microbial organic fertilizer in 2021 (Figure 5). Ks for treatments M6 to M24 decreased by 2.91% to 9.88%, indicating a significant effect of microbial organic fertilizer on altering soil physical properties. In 2022, Ks for treatments M6 to M48 decreased by 3.59% to 8.38%. In 2023, the decreasing trend in Ks became more pronounced with increasing application rates of microbial organic fertilizer, with Ks for treatments M6 to M48 decreasing by 5.95% to 13.69%. These results demonstrate that the application of microbial organic fertilizer significantly impacts soil physical properties, particularly in increasing soil organic matter content and altering the soil structure. The organic matter introduced by microbial organic fertilizer may lead to a reduction in the number of macropores and increase soil compactness, thereby reducing Ks. This effect is especially evident at higher application rates of microbial organic fertilizer due to the accumulation of organic matter and enhanced microbial activity. This phenomenon suggests that while microbial organic fertilizer increases soil organic matter content, it may also alter the soil pore structure, affecting soil water mobility and permeability [7,15].

#### 3.2.2. Soil Water-Holding Capacity

**Figure 6:**
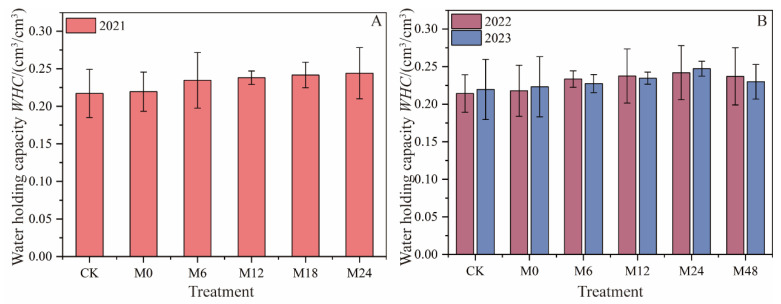
Changes in water-holding capacity under magnetically treated water drip irrigation combined with microbial organic fertilizer (**A**,**B**).

Under magnetically treated water drip irrigation with microbial organic fertilizer, the soil water-holding capacity (WHC) was significantly improved compared to conventional water irrigation (CK), and this effect increased with the application rate of microbial organic fertilizer (Figure 6). In 2021, the soil WHC for microbial organic fertilizer treatments showed a noticeable increase compared to CK (WHC = 0.217 cm^3^/cm^3^). Specifically, M6 increased the WHC to 0.235 cm^3^/cm^3^, an 8.29% rise; M12 increased the WHC to 0.238 cm^3^/cm^3^, a 9.68% rise; and higher application rates (M18 and M24) resulted in increases of 11.52% and 12.44%, respectively. This indicates a dose-dependent relationship between microbial organic fertilizer and an improved soil WHC. In 2022, the positive effects of microbial organic fertilizer remained significant, especially for M24 and M48, which showed increases of 13.08% and 10.75%, respectively. This further confirms the effectiveness of microbial organic fertilizer in enhancing the soil WHC. In 2023, M24 continued to show the highest increase at 12.27%, while the increase for M48 slightly decreased to 4.55%. This suggests that the application of microbial organic fertilizer has a long-term effect on improving the soil WHC, but application rates exceeding 2.4 t/hm^2^ may result in a negative inhibitory effect.

### 3.3. Effects of Applying Microbial Organic Fertilizer under Magnetically Treated Water Drip Irrigation on Soil Enzyme Activity

#### 3.3.1. Urease Activity

**Figure 7:**
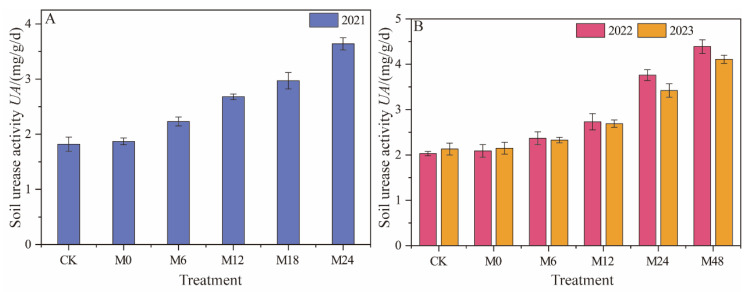
Changes in urease activity under magnetically treated water drip irrigation combined with microbial organic fertilizer (**A**,**B**).

Under magnetically treated water drip irrigation with microbial organic fertilizer, urease activity significantly increased in 2021 with the continuous increase in microbial organic fertilizer application rates, showing a trend of M0 < M6 < M12 < M18 < M24 (Figure 7). M24 exhibited the highest urease activity (3.64 mg/g/d), indicating that higher application rates of microbial organic fertilizer may more effectively promote urease activity. In 2022, the M48 treatment displayed the highest urease activity (4.39 mg/g/d), further emphasizing the effectiveness of high application rates of microbial organic fertilizer in enhancing urease activity. This result may be related to the cumulative effect of certain components in the microbial organic fertilizer or the yearly changes in soil microbial community structure. In 2023, the trend of increasing urease activity with higher microbial organic fertilizer application rates continued, following the pattern of M0 < M6 < M12 < M24 < M48. Although the urease activity of M48 (4.11 mg/g/d) was higher than that of the other treatments, it showed a slight decrease compared to 2022. This decline may point to changes in the adaptability of the soil microbial community or shifts in the equilibrium of certain biochemical processes in the soil. These data indicate that the application of microbial organic fertilizer significantly enhances soil urease activity, and this effect increases with higher application rates of microbial organic fertilizer.

In 2022 and 2023, the M48 treatment exhibited the highest urease activity, suggesting that within a certain range, higher application rates of microbial organic fertilizer more effectively promote microbial activity and urease production. With each passing year, urease activity increased for all treatment groups, possibly reflecting continuous improvements in soil conditions and increased microbial activity. This indicates the potential of microbial organic fertilizer to enhance soil urease activity. Microbial organic fertilizer may enhance urease activity by providing beneficial microbes, increasing soil organic matter content, and improving soil structure and nutrient status. The more pronounced effects observed with higher application rates of microbial organic fertilizer could be related to their greater efficacy in promoting soil microbial community diversity and functionality.

#### 3.3.2. Catalase Activity

**Figure 8:**
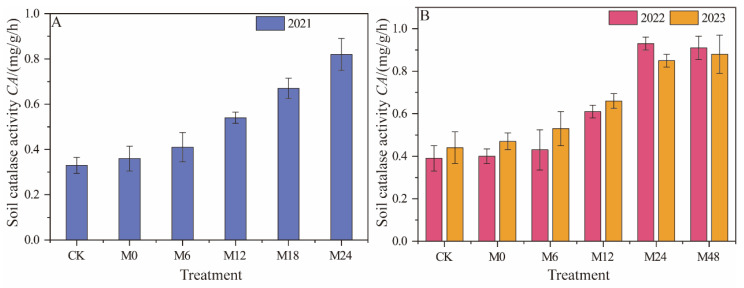
Changes in catalase activity under magnetically treated water drip irrigation combined with microbial organic fertilizer (**A**,**B**).

Under magnetically treated water drip irrigation with microbial organic fertilizer, catalase activity significantly increased in 2021 with higher application rates of microbial organic fertilizer. Notably, in the M24 treatment, catalase activity increased by 148.52%, indicating that higher application rates significantly promoted catalase activity (Figure 8). In 2022, all microbial organic fertilizer treatments showed higher catalase activity, especially M24 and M48, which increased by 138.48% and 133.30%, respectively. In 2023, the M24 (0.85 mg/g/h) and M48 (0.88 mg/g/h) treatments again exhibited high activity, with increases of 93.21% and 99.98%, respectively. The increasing trend in soil catalase activity with higher application rates of microbial organic fertilizer, particularly in high-fertilization treatments, suggests that the addition of beneficial microbes in the fertilizer improves soil biological activity and organic matter decomposition, thereby promoting catalase activity. This trend is especially pronounced at medium-to-high fertilizer application rates.

#### 3.3.3. Sucrase Activity

**Figure 9:**
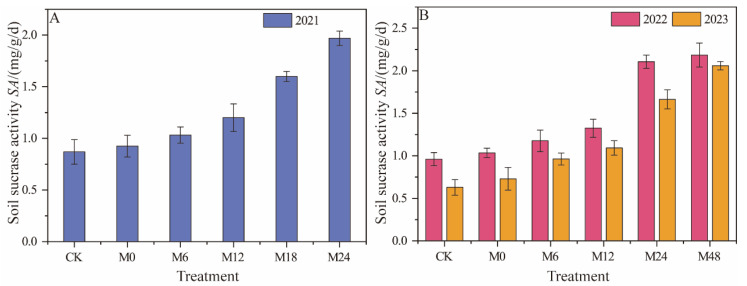
Changes in sucrase activity under magnetically treated water drip irrigation combined with microbial organic fertilizer (**A**,**B**).

Under magnetically treated water drip irrigation with microbial organic fertilizer, the application of microbial organic fertilizer significantly increased sucrase activity in 2021, with M24 showing an increase of 126.44% (Figure 9). This trend was further confirmed in 2022, where M24 and M48 exhibited the highest increases in activity, at 119.79% and 127.08%, respectively, indicating that higher application rates of microbial organic fertilizer significantly promote soil sucrase activity. In 2023, the sucrase activity of M48 increased to 2.06 mg/g/d, with a remarkable increase of 226.98%, further emphasizing the potential of microbial organic fertilizer in enhancing soil sucrase activity. These results demonstrate that the application of microbial organic fertilizer significantly enhances soil sucrase activity, and this effect increases with higher application rates. This may be due to the beneficial microbes and nutrients in the microbial organic fertilizer, which improve the activity of the soil microbial community and increase the decomposition of soil organic matter, thereby promoting the synthesis and activity of sucrase. Additionally, the year-over-year persistence of this effect highlights the long-term potential of microbial organic fertilizer in improving soil biochemical properties.

### 3.4. Effects of Applying Microbial Organic Fertilizer under Magnetically Treated Water Drip Irrigation on the Stability of Soil Aggregates

#### 3.4.1. Aggregate Composition

Table 3 shows the effect of magnetically treated water drip irrigation with microbial organic fertilizer on the composition of soil water-stable aggregates. In 2021, compared to CK, M6 significantly increased the proportion of soil aggregates larger than 0.25 mm. The 2.0–0.5 mm fraction increased from 0 to 1.63%, and the 0.5–0.25 mm fraction increased by 45.67% compared to CK. This trend was more pronounced at higher application rates (M24), with the 2.0–0.5 mm fraction increasing from 0 to 6.36% and the 0.5–0.25 mm fraction showing an increase of 142.55%. In 2022, this trend continued and intensified. The 0.5–0.25 mm fraction in the M24 treatment increased by 106.43%. This effect was even more significant in the M48 treatment, where the 0.5–0.25 mm fraction increased by 132.24%. In 2023, the 2.0–0.5 mm fraction in the M24 and M48 treatments increased by 99.08% and 133.37%, respectively, demonstrating the significant impact of microbial organic fertilizer on the soil aggregate structure. Overall, microbial organic fertilizer has the potential to improve the soil structure by promoting the formation of larger soil aggregates. This change may be due to the increased microbial activity and organic matter content provided by the microbial organic fertilizer, which enhances the binding forces between soil particles, thereby promoting the formation of large aggregates.

Overall, even without the application of microbial organic fertilizer, M0 still had some impact on the soil aggregate size distribution compared to CK. Particularly in the larger size fractions, M0 increased the proportion of aggregates, while decreasing the proportion in the smallest size fraction. This may be due to the effect of magnetically treated water irrigation on soil moisture dynamics and soil particle interactions, influencing the formation and stability of soil aggregates. However, this change was relatively minor compared to the impact of microbial organic fertilizer. Notably, in the larger size fraction (2.0–0.5 mm), microbial organic fertilizer significantly increased the proportion, indicating its role in promoting the formation of larger aggregates. This may be because microbial organic fertilizer, as a soil conditioner, enhances the binding forces between soil particles, promoting the formation and stability of larger aggregates. As the application rate of microbial organic fertilizer increased, the proportion of larger aggregates significantly rose, while the proportion of smaller aggregates correspondingly decreased. This could be related to the enhanced soil aggregation and structural stability provided by the microbial organic fertilizer. The changes in the aggregate structure improve soil aeration and water retention, thereby enhancing the soil’s biophysical environment, promoting root development, and increasing soil biological activity.

#### 3.4.2. Proportion of Water-Stable Aggregates

Under magnetically treated water drip irrigation with microbial organic fertilizer, there was a notable positive effect on soil water-stable aggregates (WSAs) in 2021 compared to CK, especially at higher application rates (Table 3). For example, the WSA for treatments M6 to M24 increased by 65.26% to 219.99% compared to CK. This significant increase indicates that microbial organic fertilizer can effectively enhance soil aggregate stability, particularly at higher application rates. In 2022, this trend was further confirmed and strengthened. The WSA for treatments M6 to M48 increased by 49.89% to 211.55%, reinforcing the positive impact of microbial organic fertilizer on soil aggregate stability. Notably, the WSA increase for M48 was the most significant, reflecting a dose–effect relationship in enhancing soil aggregate stability with microbial organic fertilizer. In 2023, microbial organic fertilizer continued to significantly improve WSAs, with increases ranging from 60.78% to 226.38% for treatments M6 to M48. The persistence and intensification of this trend may be attributed to the long-term effects of microbial organic fertilizer, particularly in providing sustained nutrients and microbial activity. Overall, the positive impact of microbial organic fertilizer on soil aggregate stability is very significant. The mechanisms may include beneficial microbes in the fertilizer breaking down organic matter to form colloidal substances that bind soil particles, promoting aggregate formation. Additionally, the organic components in the fertilizer provide a food source for soil microbes, increasing soil organic matter content and improving the water-erosion resistance of soil aggregates. The long-term application of microbial organic fertilizer improves the physical and chemical properties of the soil, promoting the formation of more stable soil aggregates. Therefore, the application of microbial organic fertilizer not only improves soil aggregate stability in the short term but also has a more pronounced effect with long-term use, highlighting its important value in sustainable soil management and agricultural practices.

### 3.5. Effects of Applying Microbial Organic Fertilizer under Magnetically Treated Water Drip Irrigation on the Yield and Quality of Jujubes

#### 3.5.1. Jujube Yield

**Figure 10:**
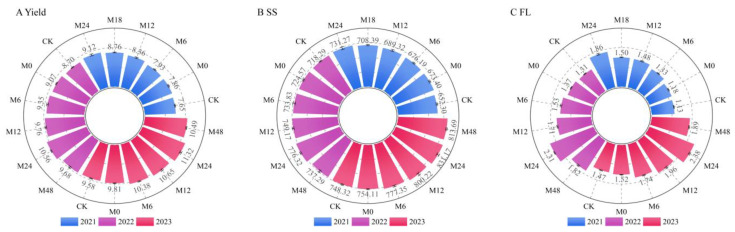
Yield (**A**), soluble sugar (**B**), and flavone (**C**) under m microbial organic fertilizer treatment by magnetically treated water drip irrigation.

Under the treatment of microbial organic fertilizer applied by magnetically treated water drip irrigation, the yield of jujubes increased significantly with the increase in microbial fertilizer applied in 2021 (Figure 10). Compared with CK, the yield of jujubes from M6 to M24 increased by 3.66% to 19.22% (from 7.65 t/hm^2^ for CK to 9.12 t/hm^2^ for M24). In 2022, the jujube yield initially increased and then decreased with the increase in microbial fertilizer application. Compared with CK, the jujube yield from M6 to M48 increased by 7.47% to 21.38% (from 8.70 t/hm^2^ for CK to 10.56 t/hm^2^ for M24 and then decreased to 9.68 t/hm^2^ for M48). In 2023, the change in jujube yield under different treatments continued the change trend of 2022, compared with CK. The jujube yield from M6 to M48 increased by 8.35% to 18.16% (from 9.58 t/hm^2^ for CK to 11.32 t/hm^2^ for M24 and then decreased to 10.49 t/hm^2^ for M48). On the whole, compared with 2022, the yield of jujubes in 2021 was lower in all treatments, and the yield of M12 in 2021 was 8.76 t/hm^2^, which was slightly higher than that of CK in 2022, 8.70 t/hm^2^. On the whole, the maximum yield of M24 was 9.12~11.32 t/hm^2^ in the three years of experiments, and the yield increase was 18.16~21.38%. Therefore, M24 can be used as a recommended measure to increase jujube yield in the desert area of southern Xinjiang. It should be noted that the application of biological fertilizer at M48 (4.8 t/hm^2^) is four times that of microbial fertilizer applied at M12 (1.2 t/hm^2^), but the output of M48 in 2022 is only 9.68 t/hm^2^, which is 0.08 t/hm^2^ less than that of M12. This indicates that the application of the appropriate microbial fertilizer can indeed play a role in increasing yield, but the increase in microbial fertilizer application may cause physiological drought of jujube roots, thus inhibiting soil respiration and nutrient conversion in the root zone. M48 is not a recommended solution in increasing jujube yield.

#### 3.5.2. Quality

Under magnetically treated water drip irrigation with microbial organic fertilizer, the proportion of premium, first-grade, and second-grade jujubes increased with higher application rates of microbial organic fertilizer in 2021, while the proportion of third-grade and below-standard dates decreased (Table 4). The proportion of premium dates increased from 8.73% in CK to 15.62% in M24, with the overall quality fruit rate improving by 80.61% (from 38.47% to 69.48%). In 2022, the proportion of premium, first-grade, and second-grade dates initially increased with higher application rates and then decreased. The proportions of premium, first-grade, and second-grade dates for treatments M6 to M48 ranged from 12.12% to 20.13%, 17.30% to 23.16%, and 26.42% to 36.33%, respectively. The overall quality fruit rate increased by 69.62% compared to CK (from 46.94% to 79.62%). In 2023, the proportions of premium dates for treatments M6 to M48 ranged from 14.32% to 23.32%, with the overall quality fruit rate increasing by 92.67% compared to CK (from 42.96% to 82.77%). The quality fruit rates for M12 and M48 exceeded 70%, while M24 further increased to 82.77%. Overall, M24 consistently achieved the highest quality fruit rate. The efficiency of M48 in improving the quality fruit rate was similar to that of M12, and M48 significantly reduced the proportion of below-standard dates in both 2022 and 2023. This indicates that an appropriate amount of microbial organic fertilizer can effectively improve the quality fruit rate of jujubes.

Under magnetically treated water drip irrigation with microbial organic fertilizer, different treatments showed varying effects on the soluble sugar content of jujubes in 2021 (Figure 10). Compared to CK, the soluble sugar content slightly increased in M0, while the increases in M6 and M12 were relatively small. However, M18 and M24 exhibited more substantial increases of 8.60% and 12.11%, respectively, indicating a significant promotion of soluble sugar content by microbial organic fertilizer in these treatments. In 2022, the changes in soluble sugar content with different microbial organic fertilizer treatments were more pronounced. Compared to CK, M0 showed a slight increase, while the increases in M6 and M12 remained small. However, the soluble sugar content in M24 significantly increased to 776.32 mg/g, an 8.08% increase compared to CK. In 2023, the soluble sugar content continued to increase across all treatments. M0 showed further increases compared to the previous two years, while the increases in M6 and M12 remained small. M24 again achieved the highest soluble sugar content at 831.17 mg/g. Notably, although the application rate of microbial organic fertilizer in M48 was twice that of M24, its soluble sugar content in 2022 and 2023 was lower than that of M24. This may be related to a counter-regulatory mechanism triggered by the excessive application of microbial organic fertilizer. First, while the soluble sugar content in M48 was slightly lower than in M24, it was still higher than in CK, indicating that the application of microbial organic fertilizer promoted the accumulation of soluble sugars in jujube fruits to some extent. Second, the performance of M48 might have been affected by the application rate; excessive microbial organic fertilizer does not necessarily result in higher sugar accumulation. This could be because the nutrient absorption capacity of jujubes is limited within a certain range, and excessive microbial organic fertilizer might lead to nutrient wastage or reach the threshold of the trees’ absorption and conversion capacity, no longer significantly promoting sugar accumulation.

Under magnetically treated water drip irrigation with microbial organic fertilizer in 2021, the flavonoid content increased significantly with higher application rates of microbial organic fertilizer. Compared to CK, which had a flavonoid content of 1.13 mg/g, M24 showed the most significant increase, reaching 1.86 mg/g, an increase of 65.49% (Figure 10). Further analysis revealed that M24 increased by 57.63% compared to M0. M12 and M18 increased by 3.33% and 6.67%, respectively, compared to the previous group, indicating a significant positive effect of gradually increasing microbial organic fertilizer on flavonoid content in jujubes. In 2022, the flavonoid content in M24 reached 2.31 mg/g, an increase of 76.34% compared to CK (1.31 mg/g). M24 increased by 51.09% compared to M0. The flavonoid content across treatments showed an initial increase followed by a decrease with increasing application rates of microbial organic fertilizer. The highest application rate, M48, showed a 21.21% decrease compared to M24 but still remained significantly higher than CK. This trend strongly supports the positive correlation between the amount of microbial organic fertilizer and the fruit quality. The results from 2023 again confirmed the significant effect of microbial organic fertilizer on the quality of jujubes. The flavonoid content in M24 reached a maximum of 2.38 mg/g, an increase of 62.59% compared to CK (1.47 mg/g) and a 56.58% increase compared to M0. However, M48 showed a 20.67% decrease compared to M24. Over the three years, the results consistently showed that increasing microbial organic fertilizer significantly enhanced the flavonoid content in jujubes. M24 consistently performed the best, with an average increase of 68.81% compared to CK. It is noteworthy that the differences in increases between different treatment groups gradually decreased over time. This suggests that there may be a saturation point in the effect of microbial organic fertilizer on flavonoid content within a certain range. Nevertheless, the effect of microbial organic fertilizer persists across different application rates, continuously enhancing the flavonoid content in jujubes.

### 3.6. Quantitative Characterization of Gauss Model

#### 3.6.1. Prediction of Optimal Application Amount of Microbial Organic Fertilizer Based on Soil Pore Volume, Urease Activity, and WSA

**Figure 11:**
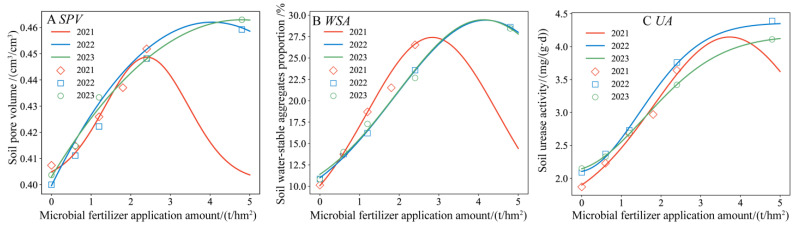
The relationship between soil pore volume (**A**), the proportion of water-stable aggregates (**B**), and urease activity (**C**) predicted by the model and measured values.

As shown in Figure 11 and Table 5, the Gauss model effectively predicted the soil property indices SPV, WSA, and UA under magnetically treated water combined with different application rates of microbial organic fertilizer. The R^2^ values between the predicted and measured values ranged from 0.936 to 0.999. Overall, the optimal application rate for microbial organic fertilizer corresponding to SSWCmax varied slightly across different years, ranging from 2.14 to 3.03 t/hm^2^. The SSWCmax values were relatively close, ranging from 0.406 to 0.414 cm^3^/cm^3^. The optimal application rates for SPVmax, WSAmax, and UAmax in 2021 were lower than in 2022 and 2023, likely because the maximum application rate of microbial organic fertilizer in 2021 was only 2.40 t/hm^2^, causing the Gauss model to predict a lower optimal rate. In 2022 and 2023, the maximum application rate was increased to 4.80 t/hm^2^, improving the precision of the Gauss model predictions. Except for UAmax in 2023, the optimal application rates for the other indices in 2022 and 2023 ranged from 3.89 to 5.13 t/hm^2^. From an economic perspective, excluding the higher application rates of 5.86 and 5.13 t/hm^2^ and averaging the optimal rates for the other indices gives an approximate optimal rate of 3.47 t/hm^2^. Therefore, from the perspective of comprehensively improving desert soil properties, an application rate of 3.47 t/hm^2^ is recommended for microbial organic fertilizer. While applying more microbial organic fertilizer can further significantly enhance soil nutrients and microbial activity, excessive application can directly increase production costs and potentially cause soil acidification and environmental pollution.

#### 3.6.2. Construction of Jujube Yield and Quality Prediction Model Based on Microbial Organic Fertilizer Application Amount

**Figure 12:**
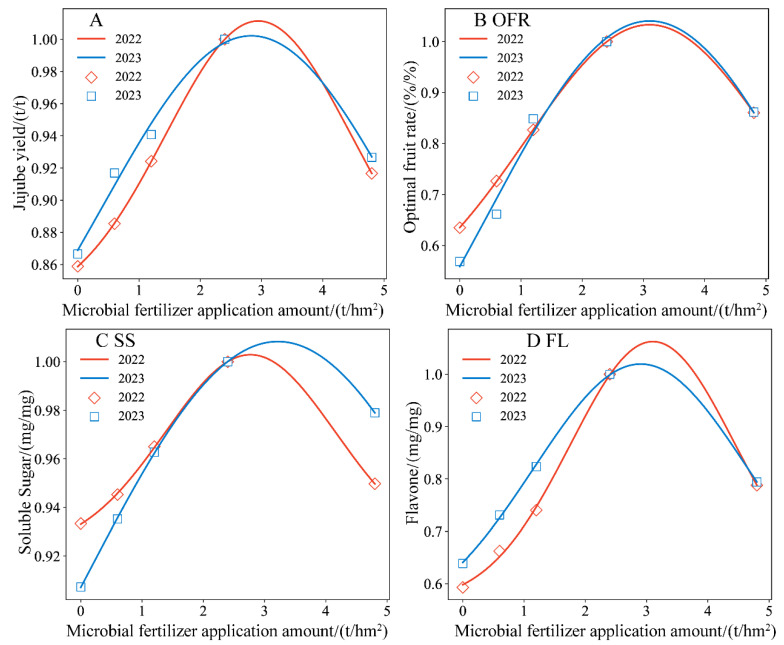
The prediction curve and measured value of the jujube yield and quality with the application of microbial fertilizer with the Gauss model (**A**–**D**).

In Figure 12 and Table 6, the Gauss model curves accurately fitted the jujube indices under different application rates of microbial organic fertilizer, with R^2^ ≥ 0.854 and RMSE ≤ 0.0333. The normalized target values for the microbial organic fertilizer treatments were relatively close, and the peak or trough values and their corresponding optimal application rates were consistent across different years for the same index. For the yield target, the optimal application rates of microbial organic fertilizer were 2.95 and 2.83 t/hm^2^. For the SS target, the optimal application rates were 2.77 and 3.22 t/hm^2^. For the FL target, the optimal application rates were 3.10 and 2.90 t/hm^2^. Overall, the optimal application rate range for microbial organic fertilizer was 2.77 to 3.22 t/hm^2^. Averaging these optimal rates gives a value of 3.09 t/hm^2^. Therefore, 3.09 t/hm^2^ is recommended as the optimal application rate of microbial organic fertilizer to comprehensively improve the yield and quality and reduce water consumption.

To further quantitatively analyze the effectiveness of microbial organic fertilizer application on the comprehensive quality of jujubes under magnetically treated water drip irrigation, we hypothesized *ξ* as the magnetization efficiency coefficient. Using the 2022 experimental data, we derived the Gauss model parameters and constructed a predictive model for jujube yield and quality as a function of microbial organic fertilizer application rates. This predictive model was then validated using the 2023 experimental data. The specific model is as follows:

(3) $$\frac{Yield}{Yield_{\max}}=0.227\xi \exp\left(-\frac{{\left(X-2.890\right)}^{2}}{7.887}\right)+0.780\xi$$

(4) $$\frac{OFR}{OFR_{\max}}=0.784\xi \exp\left(-\frac{{\left(X-3.098\right)}^{2}}{11.080}\right)+0.253\xi$$

(5) $$\frac{SS}{SS_{\max}}=0.167\xi \exp\left(-\frac{{\left(X-2.993\right)}^{2}}{10.440}\right)+0.839\xi$$

(6) $$\frac{FL}{FL_{\max}}=0.502\xi \exp\left(-\frac{{\left(X-3.003\right)}^{2}}{4.771}\right)+0.539\xi$$

**Figure 13:**
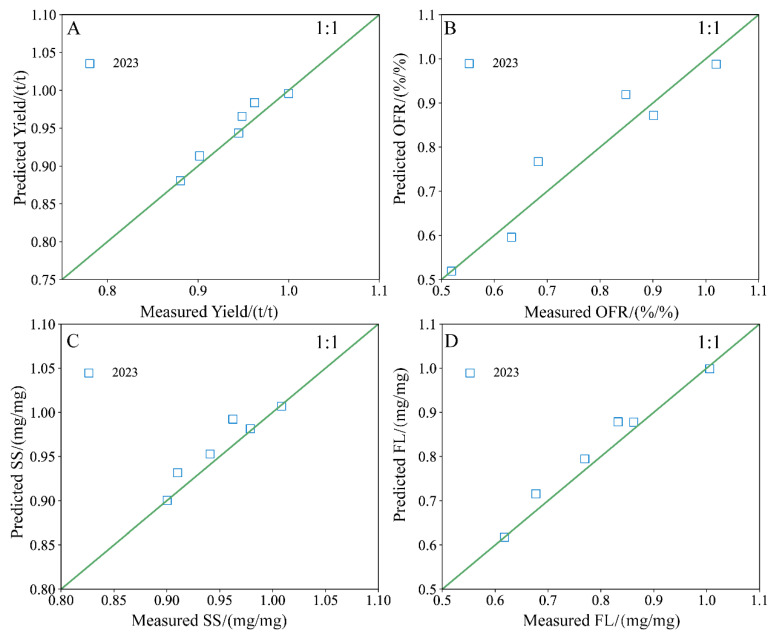
The relationship between the predicted value and the measured value of yield and quality of jujubes applied with microbial fertilizer under magnetoelectric activated water drip irrigation (**A**–**D**).

To verify the accuracy and applicability of the comprehensive predictive model, we compared the model’s calculated values with the measured values. As shown in Figure 13, the data points are distributed on both sides of the 1:1 line. Overall, the model effectively predicted the jujube yield, quality, and water consumption under magnetically treated water drip irrigation with microbial organic fertilizer, with R^2^ ≥ 0.765.

Specifically, the synergistic effects of magnetically treated water and microbial organic fertilizer are mainly realized through the following mechanisms: Magnetically treated water changes the structure of water molecules, increasing the activity and permeability of water and enhancing dissolved oxygen content, thereby providing a better living environment for soil microorganisms and promoting their growth and metabolic activities [29]. Magnetically treated water can break the hydrogen bonds of water molecules, making the molecular clusters smaller, thus increasing the solubility and diffusion capacity of water, allowing for water and nutrients to distribute more quickly and evenly in the soil [29].

Microbial organic fertilizer decomposes organic matter, releasing nutrients available to plants and improving the soil structure, thereby enhancing soil aeration and water retention [27]. The beneficial microbial communities in the organic fertilizer can decompose organic substances through biological metabolic activities, releasing essential elements such as nitrogen, phosphorus, and potassium and synthesizing various secondary metabolites that promote plant growth, such as plant growth regulators and antibiotics [19]. Moreover, microbial organic fertilizer effectively promotes the formation of soil aggregates, enhancing the water and nutrient retention capacity of desert soils and reducing the number of pathogenic bacteria through competitive exclusion and antagonistic actions [23].

Under the synergistic action of both, magnetized water increases the solubility of nutrients, making the nutrients released from the decomposition of organic matter by microorganisms more easily absorbed by the jujube tree [27]. This also enhances the jujube trees’ resistance to pests, diseases, and adverse conditions, thereby promoting healthy growth. This comprehensive effect is confirmed in the previous research results by the increased microbial activity, enzyme activity, improvement in soil physical structure, and growth indicators such as the yield and quality of jujubes [27].

Overall, the model constructed in this study effectively predicted the yield and quality of jujubes under magnetically treated water drip irrigation with the application of microbial organic fertilizer, achieving a high degree of simulation accuracy. This provides theoretical support and model backing for high-yield and high-quality jujube production in the sandy areas of Xinjiang. However, it should be noted that the model constructed in this study still has several limitations. Although we optimized the balance between model simplification and accuracy, the model did not account for the effects of varying climatic conditions across different years on yield and quality. As a result, there are certain differences in the yield and quality between different years. While normalization was used to minimize the impact of environmental factors, some discrepancies between years remained, leading to a lower goodness-of-fit for the predictive model.

Microbial organic fertilizers have been proven to be more effective than chemical fertilizers. The growth-promoting effect of microbial organic fertilizers may be due to the increased root activity in the rhizosphere and the activation of hormones, thereby enhancing nutrient uptake by plants [12]. Several studies have reported the beneficial effects of biofertilizers on plant growth. The application of Bacillus subtilis microbial organic fertilizer effectively delayed the coloration of the peel and flesh of blood oranges, increased the nitrogen and potassium concentrations in leaves, and reduced titratable acidity (TA) [22]. Additionally, Bacillus subtilis microbial organic fertilizer improved the yield per tomato plant, marketable yield, fruit weight, and length and enhanced tomato quality [23]. Our study also obtained similar results, with the jujube yield increasing by 3.66% to 21.38%, a significant reduction in titratable acidity, and notable increases in soluble sugar and flavonoid contents, with the sugar–acid ratio improving by more than 40%. This is attributed to the application of microbial organic fertilizer, which enhanced the water and nutrient retention capacity of desert soil, inhibited deep water and nutrient leaching, and provided adequate water and nutrient support for the growth of jujube trees. Moreover, microbial organic fertilizer increased soil enzyme activity, promoting metabolic processes such as sugar breakdown and organic matter synthesis in plants, thereby increasing the synthesis of soluble sugars and flavonoids [12]. It is noteworthy that the correlation analysis results indicated that the improvement in soil moisture content and enzyme activity were the main factors leading to the increase in jujube yield. Additionally, our study found that microbial organic fertilizer significantly improved irrigation water use efficiency and the partial factor productivity of N, P, and K fertilizers [27]. This is likely due to the microbial organic fertilizer reducing soil saturated hydraulic conductivity and increasing soil porosity, while microorganisms such as Bacillus subtilis in the fertilizer accelerated nutrient conversion and absorption, thereby enhancing the jujube yield [27].

## 4. Conclusions

This study investigated the effects of magnetically treated water drip irrigation combined with microbial organic fertilizer on soil physical properties, hydraulic parameters, enzyme activity, aggregate stability, and the yield and quality of jujubes. The results indicated that the application of microbial organic fertilizer significantly reduced the soil bulk density, increased soil porosity, improved the soil water-holding capacity and enzyme activity, and increased the proportion of large aggregates. These improvements were more pronounced with higher application rates of microbial organic fertilizer. High application rates showed more significant effects on various soil indices, demonstrating the important potential of microbial organic fertilizer in improving soil structure, enhancing soil fertility, and promoting plant growth. Additionally, the combination of magnetically treated water drip irrigation and microbial organic fertilizer significantly increased the yield and quality of jujubes. The Gauss model calculation recommended an optimal application rate of 3.09 t/hm^2^ to comprehensively improve the yield and quality of jujubes. This study provides scientific evidence for soil improvement and agricultural production, offering important practical guidance.

## Figures and Tables

**Table 1:** Physical and Chemical Characteristics of Soil Profile.

Soil Depth (cm)	Sand (%)	Silt (%)	Clay (%)	Soil Texture	Soil Bulk Density (g/cm^3^)	pH	Fast-Acting Potassium (mg/kg)	Fast-Acting Phosphorus (mg/kg)
0–20	86.77	13.20	0.03	Sand	1.62	8.3	25.31	12.31
20–40	86.68	13.14	0.18	Sand	1.61	8.2	22.14	8.29
40–60	85.49	12.33	2.18	Sand	1.59	8.3	20.11	6.52
60–80	85.29	12.47	2.24	Sand	1.60	8.4	15.18	4.87

**Table 2:** Irrigation and fertilization system of jujube trees in 2021, 2022, and 2023.

Year	Irrigation Time	Irrigation Amount (mm)	Urea (kg/ha)	P_2_O_5_ (kg/ha)	K_2_O (kg/ha)
2021	20 April	32	37.95	18.90	6.48
	5 May	32	37.95	18.90	6.48
	20 May	32	37.95	18.90	6.48
	3 June	32	37.95	18.90	6.48
	17 June	32	37.95	18.90	6.48
	2 July	32	43.20	14.40	38.40
	15 July	32	43.20	14.40	38.40
	1 August	32	43.20	14.40	38.40
	16 August	32	43.20	14.40	38.40
	2 September	32	43.20	14.40	38.40
2022	28 April	32	37.95	18.90	6.48
	13 May	32	37.95	18.90	6.48
	28 May	32	37.95	18.90	6.48
	10 June	32	37.95	18.90	6.48
	25 June	32	37.95	18.90	6.48
	8 July	32	43.20	14.40	38.40
	21 July	32	43.20	14.40	38.40
	2 August	32	43.20	14.40	38.40
	18 August	32	43.20	14.40	38.40
	3 September	32	43.20	14.40	38.40
2023	27 April	32	37.95	18.90	6.48
	8 May	32	37.95	18.90	6.48
	20 May	32	37.95	18.90	6.48
	3 June	32	37.95	18.90	6.48
	16 June	32	37.95	18.90	6.48
	1 July	32	43.20	14.40	38.40
	15 July	32	43.20	14.40	38.40
	29 July	32	43.20	14.40	38.40
	13 August	32	43.20	14.40	38.40
	28 August	32	43.20	14.40	38.40

**Table 3:** Effects of microbial fertilizer treatment applied by magnetoelectric activated water drip irrigation on soil aggregate particle size composition in 2021, 2022, and 2023.

Year	Treatment	Soil Aggregate Size Distribution (%)			
		2–0.5 mm	0.5–0.25 mm	0.25–0.125 mm	<0.125 mm
2021	CK	0	8.32 e	21.72 f	69.96 a
	M0	0	10.13 de	26.01 d	63.86 b
	M6	1.63 e	12.12 d	28.27 c	57.98 c
	M12	3.88 d	14.82 c	28.61 c	52.69 d
	M18	4.76 c	16.76 b	28.22 c	50.26 e
	M24	6.36 b	20.18 a	31.33 a	42.13 g
2022	CK	0	9.18 e	23.21 e	67.61 a
	M0	0	10.85 d	25.17 d	63.98 b
	M6	1.93 e	11.83 d	27.32 cd	58.92 c
	M12	2.52 d	13.69 c	28.63 c	55.16 d
	M24	4.63 c	18.95 b	30.14 ab	46.28 f
	M48	7.28 ab	21.32 a	33.16 a	38.24
2023	CK	0	8.72 e	24.21 de	67.07 a
	M0	0	10.83 d	25.47 d	63.70 b
	M6	1.91 e	12.11 d	26.25 d	59.73 c
	M12	2.95 d	14.34 c	29.15 c	53.56 d
	M24	5.31 b	17.36 bc	31.48 ab	45.85 f
	M48	8.11 a	20.35 a	34.37 a	37.17 h

Different lowercase letters above bars indicate significant differences at *p* < 0.05.

**Table 4:** Jujube grading and the optimal fruit rate under the treatment of microbial fertilizer applied by magnetoelectric activated water drip irrigation.

Year	Treatment	Percentage of Jujube Fruit Grades (%)					Optimal Fruit Rate (%)
		Special	Level 1	Level 2	Level 3	Outside the Level	
2021	CK	8.73 g	12.13 g	17.61 g	33.14 a	28.39 a	38.47 j
	M0	9.65 f	15.12 e	16.77 g	30.18 b	28.28 a	41.54 i
	M6	10.38 f	16.35 e	18.22 f	29.16 b	25.89 a	44.95 h
	M12	11.52 e	17.62 d	21.62 e	27.24 c	22.00 c	50.76 f
	M18	12.66 e	18.84 c	27.67 c	23.17 d	17.66 d	59.17 d
	M24	15.62 d	22.18 b	31.68 b	21.22 e	9.30 g	69.48 b
2022	CK	10.34 f	15.40 e	21.20 e	27.60 c	25.46 b	46.94 g
	M0	12.12 e	14.13 e	24.31 d	23.87 d	25.57 b	50.56 f
	M6	14.12 d	17.30 d	26.42 c	23.19 d	18.97 d	57.84 d
	M12	15.31 d	19.14 c	31.39 b	20.11 e	14.05 f	65.84 c
	M24	20.13 b	23.16 b	36.33 a	13.35 f	7.03 h	79.62 a
	M48	14.88 d	20.36 c	33.27 b	23.36 d	8.13 h	68.51 b
2023	CK	11.33 f	14.28 f	17.35 g	37.32 a	19.72 d	42.96 i
	M0	12.51 e	15.34 e	19.22 f	36.79 a	16.14 e	47.07 g
	M6	14.32 d	17.27 d	23.17 d	30.76 b	14.48 f	54.76 e
	M12	17.31 c	20.33 c	32.61 b	22.66 d	7.09 h	70.25 b
	M24	23.32 a	26.17 a	33.28 b	12.69 f	4.54 i	82.77 a
	M48	16.73 c	19.33 c	35.27 a	23.28 d	5.39 i	71.33 b

Different lowercase letters above bars indicate significant differences at *p* < 0.05.

**Table 5:** Soil property factor prediction model parameters and evaluation indexes under microbial fertilizer treatment for magneto-electric activated water drip irrigation in 2021, 2022, and 2023.

Year	Index	Optimal Application Amount	Optimal Index Value	R^2^	RMSE
2021	SPV	2.40	0.45	0.968	0.003
	WSA	2.83	27.41	0.999	0.096
	UA	3.73	4.15	0.991	0.066
2022	SPV	3.89	0.46	0.936	0.006
	WSA	4.18	29.43	0.999	0.207
	UA	5.13	4.51	0.996	0.085
2023	SPV	4.60	0.46	0.992	0.002
	WSA	4.13	29.47	0.968	0.356
	UA	5.86	4.33	0.998	0.057

**Table 6:** The parameters and evaluation indexes of the optimal yield and quality prediction (Gauss) model under the microbial fertilizer treatment of magneto-electric activated water drip irrigation.

Year	Index	*a*	*x*_0_	*β*	*TV*_0_	*TV*_0_ + *a*	R^2^	RMSE
2022	Yield	0.180	2.947	1.513	0.832	1.011	0.999	0.0002
	OFR	0.574	3.096	2.014	0.459	1.033	0.999	0.0006
	TA	−0.325	3.330	1.867	1.012	0.687	0.854	0.0333
	SS	0.081	2.769	1.383	0.922	1.003	0.999	0.0003
	FL	0.499	3.102	1.344	0.563	1.063	0.998	0.0061
	ET	−0.090	3.160	1.768	1.012	0.922	0.999	0.0008
2023	Yield	0.275	2.833	2.459	0.728	1.002	0.987	0.0048
	OFR	0.995	3.100	2.693	0.046	1.040	0.984	0.0192
	TA	−1.800	3.273	5.329	2.458	0.658	0.999	0.0012
	SS	0.253	3.218	3.186	0.755	1.008	0.999	0.0003
	FL	0.505	2.904	1.745	0.514	1.020	0.999	0.0033
	ET	−0.077	3.297	1.340	0.998	0.921	0.999	0.0001

## Data Availability

The data related to the research are reported in the paper. Any additional data may be acquired from the first corresponding author upon request.
